# FAIR‐compliant clinical, radiomics and DICOM metadata of RIDER, interobserver, Lung1 and head‐Neck1 TCIA collections

**DOI:** 10.1002/mp.14322

**Published:** 2020-06-27

**Authors:** Petros Kalendralis, Zhenwei Shi, Alberto Traverso, Ananya Choudhury, Matthijs Sloep, Ivan Zhovannik, Martijn P.A. Starmans, Detlef Grittner, Peter Feltens, Rene Monshouwer, Stefan Klein, Rianne Fijten, Hugo Aerts, Andre Dekker, Johan van Soest, Leonard Wee

**Affiliations:** ^1^ Department of Radiation Oncology (Maastro) GROW School for Oncology Maastricht University Medical Centre+ Maastricht 6229 ET The Netherlands; ^2^ Department of Radiation Oncology Radboud University Medical Center Nijmegen 6525 GC The Netherlands; ^3^ Department of Radiology and Nuclear Medicine Erasmus Medical Center Rotterdam 3015 GD The Netherlands; ^4^ Department of Medical Informatics Erasmus Medical Center Rotterdam 3015 GD The Netherlands; ^5^ SOHARD Software GmbH Fuerth 90766 Germany; ^6^ Artificial Intelligence in Medicine (AIM) Program Brigham and Women’s Hospital Harvard Medical School Boston MA 02115 United States; ^7^ Radiology and Nuclear Medicine CARIM & GROW School for Oncology Maastricht University Maastricht 6211 LK The Netherlands

**Keywords:** datasets, FAIR, radiomics, repeatability, reproducibility

## Abstract

**Purpose:**

One of the most frequently cited radiomics investigations showed that features automatically extracted from routine clinical images could be used in prognostic modeling. These images have been made publicly accessible via The Cancer Imaging Archive (TCIA). There have been numerous requests for additional explanatory metadata on the following datasets — RIDER, Interobserver, Lung1, and Head–Neck1. To support repeatability, reproducibility, generalizability, and transparency in radiomics research, we publish the subjects’ clinical data, extracted radiomics features, and digital imaging and communications in medicine (DICOM) headers of these four datasets with descriptive metadata, in order to be more compliant with findable, accessible, interoperable, and reusable (FAIR) data management principles.

**Acquisition and validation methods:**

Overall survival time intervals were updated using a national citizens registry after internal ethics board approval. Spatial offsets of the primary gross tumor volume (GTV) regions of interest (ROIs) associated with the Lung1 CT series were improved on the TCIA. GTV radiomics features were extracted using the open‐source Ontology‐Guided Radiomics Analysis Workflow (O‐RAW). We reshaped the output of O‐RAW to map features and extraction settings to the latest version of Radiomics Ontology, so as to be consistent with the Image Biomarker Standardization Initiative (IBSI). Digital imaging and communications in medicine metadata was extracted using a research version of Semantic DICOM (SOHARD, GmbH, Fuerth; Germany). Subjects’ clinical data were described with metadata using the Radiation Oncology Ontology. All of the above were published in Resource Descriptor Format (RDF), that is, triples. Example SPARQL queries are shared with the reader to use on the online triples archive, which are intended to illustrate how to exploit this data submission.

**Data format:**

The accumulated RDF data are publicly accessible through a SPARQL endpoint where the triples are archived. The endpoint is remotely queried through a graph database web application at http://sparql.cancerdata.org. SPARQL queries are intrinsically federated, such that we can efficiently cross‐reference clinical, DICOM, and radiomics data within a single query, while being agnostic to the original data format and coding system. The federated queries work in the same way even if the RDF data were partitioned across multiple servers and dispersed physical locations.

**Potential applications:**

The public availability of these data resources is intended to support radiomics features replication, repeatability, and reproducibility studies by the academic community. The example SPARQL queries may be freely used and modified by readers depending on their research question. Data interoperability and reusability are supported by referencing existing public ontologies. The RDF data are readily findable and accessible through the aforementioned link. Scripts used to create the RDF are made available at a code repository linked to this submission: https://gitlab.com/UM‐CDS/FAIR‐compliant_clinical_radiomics_and_DICOM_metadata.

## 1 INTRODUCTION

Clinical radiological imaging, such as computed tomography (CT), is a mainstay modality for diagnosis, screening, intervention planning, and follow‐up for cancer patients worldwide.^1^ Radiomics refers to high‐throughput automated characterization of the tumor phenotype by analyzing quantitative features derived from a radiological image.^2^ Aerts et al. showed that CT radiomics features by themselves could contain information that is potentially prognostic of overall survival in nonsmall cell lung (NSCLC) and head‐and‐neck (HN) cancer.^3^ The radiomics hypothesis is that computationally derived features extract more information than can be processed by an unaided human eye, and therefore offers up new image biomarkers to speed up the research of personalized medicine. Radiomics has the potential to be a highly cost‐effective option for retrospective observational clinical studies, since it can process routinely collected clinical radiological images residing in institutional archives. There remain significant challenges in regards to developing generalizable models that are based on reproducible and repeatable radiomics signatures.^4^, ^5^, ^6^, ^7^ Recent studies have suggested that harmonization of radiomics features across multiple institutions and different scanner parameters may be needed to realize its full potential.^8^, ^9^, ^10^, ^11^

Computed tomography images for some frequently cited studies,^3^, ^12^ in the digital imaging and communications in medicine (DICOM) format, have been made available via The Cancer Imaging Archive (TCIA).^12^, ^13^, ^14^, ^15^, ^16^ The DICOM standard incorporates metadata about image acquisition settings and it extends to regions of interest (ROIs) delineations (i.e., radiotherapy structure set, or RTSTRUCT), but many nonradiology researchers remain unfamiliar with this conjoined data‐metadata format. Pixel data only formats such as Neuroimaging Informatics Technology Initiative (NIfTI) and Nearly Raw Raster Data (NRRD) may be more intuitive for direct computation, but these have been stripped of imaging metadata. Imaging metadata is the essential context to understand why radiomics features from different scanners may or may not be reproducible.^17^, ^18^, ^19^, ^20^ Software libraries are available that easily change from DICOM to NIfTI/NRRD,^21^ but in keeping with FAIR (Findable, Accessible, Interoperable, and Reusable) data stewardship principles,^22^ the imaging metadata needs to be preserved in such a way that links to the source images and postacquisition analyses will be retained.

A similar argument holds for patients’ clinical metadata and extracted radiomics features. Publishing tables of values as open access data does not by itself comply with FAIR principles, because there may be no metadata that richly describe what the data fields are, what its contents signify, and how it relates to other data. The point of FAIR principles is not only humans should grasp enough context about the data to use it meaningfully, but that the data must be made amenable for machine algorithms to automatically search and process, even on a massive global scale.

Consider an example specific to radiomics. For a given feature, it is essential to describe how this feature is uniquely defined, which radiomics software (and version) was used to extract it, and what (if any) digital image preprocessing had been applied prior to extraction. Semantic ontologies^23^ were developed in order to add descriptive metadata and hierarchical relationships on top of the data. Ontologies make explicit the formal meaning of concepts within its proscribed domain and the essential relationships between its set of concepts. The present work reuses the Radiation Oncology Ontology (ROO),^24^ Semantic DICOM ontology (SeDI),^25^ and the radiomics ontology (RO).^26^ These ontologies themselves reuse existing terminologies and thesauri, such as the image biomarker standardization initiative (IBSI),^27^ National Cancer Institute Thesaurus (NCIT),^28^ the units of measurement ontology (UO),^29^ and the DICOM data dictionary,^30^ to identify its concepts.

Other advantages of ontologies include knowledge representation and the support for automated logical inferencing. A hierarchical structure is abstracted as directed acyclic graphs, wherein concepts and relationships are represented as vertices and edges of the graph, respectively. Any graph, regardless of complexity, can be written out in full as a series of machine‐readable sentences consisting of strictly three pieces; subject (start vertex) — predicate (edge) — object (end vertex). Such “triples” are the basis of the resource descriptor format (RDF) that is a type of universal data storage format on the World Wide Web. Machine‐based data mining and inferencing tasks are thus feasible in a highly efficient manner, being simplified to a “pattern matching” problem.

The objective of this open data submission is to stimulate studies into repeatability, reproducibility, replication, and reusability of radiomics features from multiple datasets. The core collection being made publicly available here consists of (a) improvements to the four clinical imaging datasets described in the seminal radiomics publication by Aerts et al.,^3^ (b) extracted radiomics features described in line with IBSI recommendations,^27^, ^31^ and (c) updates to the subject clinical data associated with the aforementioned image collections.

## 2 ACQUISITION AND VALIDATION METHODS

### 2.A. Description of the dataset

The metadata published in this submission links to four image collections, available under a Creative Commons license (Attribution‐NonCommercial Unported; CC BY‐NC 3.0^12^), in DICOM format on TCIA and has been previously investigated by Aerts et al.^3^. These collections are described in detail elsewhere; a brief recapitulation is given in Table I.

In each of these collections, primary Gross Tumor Volumes (GTVs) had been delineated by experienced radiation oncologists; ROIs are included in the TCIA collections as RTSTRUCT and SEGMENTATION objects. In the original TCIA submission, some ROIs were vertically displaced due to the how treatment couch offsets were being reported by legacy radiotherapy treatment planning software – these have now been corrected.

Clinical data have been extracted from patients’ electronic medical records and, where applicable, survival intervals from commencement of radiotherapy treatment till date of death or loss to follow‐up were updated using a national registry after internal review board approval. The clinical data have been made available with the imaging collections on TCIA.

### 2.B. Data format and usage notes

**Fig. 1:**
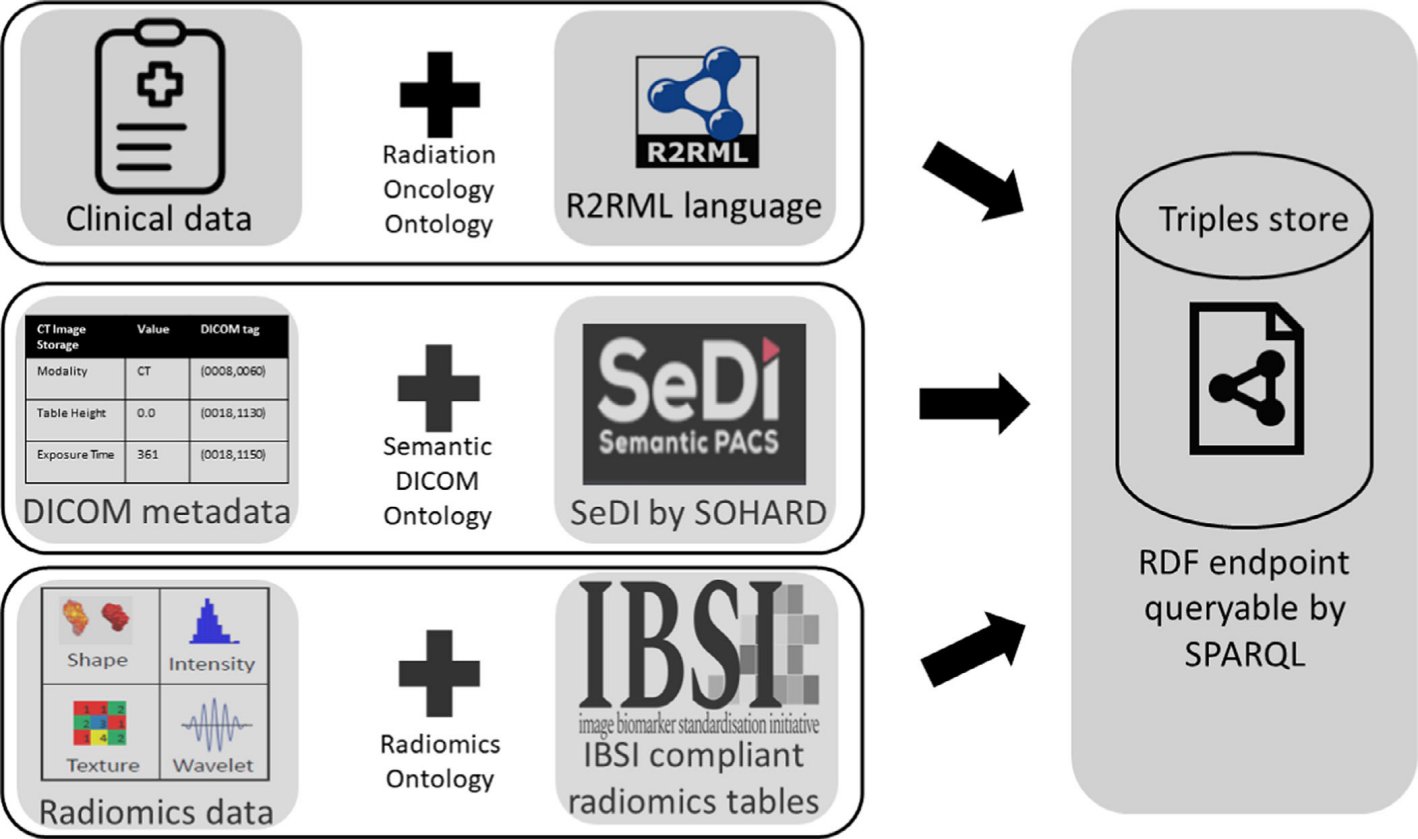
Representation of the conversion of the clinical data, digital imaging, and communications in medicine headers and radiomics features to resource descriptor format (RDF). The procedures are outlined in the text in sections 2B2, 2B3, and 2B4. The RDF triples can be queried from a publicly accessible endpoint using the SPARQL language. [Color figure can be viewed at wileyonlinelibrary.com]

The workflow of the conversion of clinical data, DICOM metadata, and radiomics features to RDF triples is represented in Fig. 1.

#### 2.B.1. Clinical metadata as RDF

Clinical tables (in CSV format) from TCIA were imported as standard relational databases (e.g., in PostGreSQL)^32^ and then converted into RDF triples using a serializing scripting language such as R2RML.^33^ R2RML allows the expression of an arbitrary relational database as an equivalent graph data object using a suitable target ontology (in this case, the ROO) which can be controlled by specifying a mapping file. The values of, and relationships between, the clinical data concepts were mapped onto a graph structure. A visual representation of an example ROO graph has been given by Traverso et al.^24^ The graph was exported as RDF triples and archived on a publicly query‐able SPARQL endpoint. The mapping files used for the RDF triples acquisition in this particular data submission are made available for the reader on a public GitLab repository https://gitlab.com/UM‐CDS/FAIR‐compliant_clinical_radiomics_and_DICOM_metadata. [Correction added on September 3, 2020, after first online publication: The referenced URL have been corrected.]

#### 2.B.2. DICOM metadata as RDF

The DICOM headers present in the abovementioned TCIA image collections were processed into graph objects using SeDI as the target ontology. A research‐only version of the Semantic DICOM conversion service of SOHARD GmbH (Fuerth, Germany) was used to automatically extract the headers from DICOM files and subsequently export these as RDF triples to the same aforementioned SPARQL endpoint. This semantic representation of imaging metadata supports cross‐referenced queries of DICOM tags against radiomics features for use in repeatability and reproducibility studies.^34^

#### 2.B.3. Radiomics metadata as RDF

The radiomics feature values of the primary GTV in the abovementioned image collections were extracted using the Ontology‐Guided Radiomics Analysis Workflow (O‐RAW),^35^ a PyRadiomics^36^ — based FAIR‐ification tool. Acquisition of the radiomics RDF triples required a two‐stage process. The results of a radiomics extraction software application (in our case O‐RAW, but the same holds for other software) must first be transferred into a set of inter‐related tables needed for the IBSI. For this submission, we prepared a python script to fill these tables more efficiently; this is provided as an example for the reader on the repository https://gitlab.com/UM‐CDS/FAIR‐compliant_clinical_radiomics_and_DICOM_metadata. [Correction added on September 3, 2020, after first online publication: The referenced URL have been corrected.] Details of radiomics ontology development and its integration with the IBSI exceed the scope of this data article, but will be covered in detail in a separate publication.^37^ Radiomics RDF triples were saved to the same aforementioned SPARQL endpoint.

### 2.C. SPARQL public endpoint

The SPARQL query language is used to interrogate the clinical, DICOM, and radiomics triples that are archived in RDF as a publicly accessible internet resource referred to by the Universal Resource Locator (URL), http://sparql.cancerdata.org/. The RDF triples are maintained in a persistent online graph database through a Blazegraph^38^ software application, which also supplies a user interface through which remote SPARQL queries may be entered. A public query may be executed as follows: after accessing the above URL, the *Namespaces* tab is selected and *Nat_Com_Collections_final* database is set to use. Queries may then be typed by hand or copy‐pasted in the *Query* tab.

### 2.D. Example SPARQL queries

The first hypothetical example we consider is a researcher who wishes to get the data for a univariate model for overall survival in the Lung1 collection, such as Welch et al.,^39^ using a single radiomics feature that is known by its IBSI text label “Fmorph.vol.” We have setup the example query in Box 1. In brief, a SPARQL query consists of:

- Shorthand prefixes for namespaces referring to data, schema, syntax, and ontologies that are needed;
- SELECT and FILTER commands that allow us to shape the contents to be returned; and,
- a sequence of pattern matching rules that allow us to link patients to radiomics features and overall survival outcome.

The contents of Box 1 may be copied and pasted into the query window of Blazegraph (http://sparql.cancerdata.org/#query). Note that a patient study identifier links both the radiomics and clinical triples, such that we can query into both domains and cross‐reference them within a single SPARQL query. The result of this example query that is limited (for display purposes) to ten subjects can be seen in Fig. 22.

**Fig. 2.**
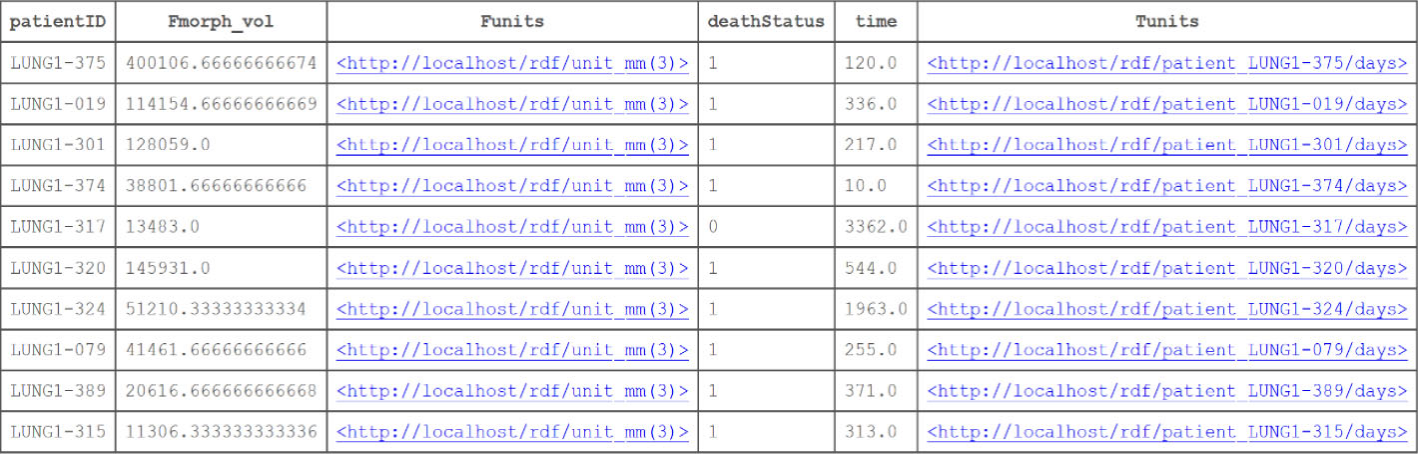
The result of ten patients’ cases of the example query given in Box 1. We can see the research study IDs of patients from the public The Cancer Imaging Archive collections, the value of a radiomics feature, the value of the survival time, and the vital status of each patient. Additionally, we have displayed the units of the radiomics feature (if any, in this case it is cubic millimeters) and the survival time (days). [Color figure can be viewed at wileyonlinelibrary.com]

As another purely radiomics‐based example, we may examine if distinct radiomics intensity discretization algorithms had been used during the extraction of a radiomics feature. If one were to execute the example query in Box 2, it would be seen that the specific radiomics feature labeled as RO:Y1RO^40^ had been computed with 12 unique feature extraction settings, but only three discretization settings were used, all of which employed a fixed bin size (FBS) method.

In our final example, we bring elements of the previous examples together into a single SPARQL query that cross‐references DICOM, radiomics, and clinical follow‐up. In the example provided in Box 3, we index the imaging modality (CT) with its Series Instance UID and Slice Thickness to the subset of morphological (ROI‐dependent) radiomics features that were computed for the Lung1 dataset, along with the corresponding survival time and survival status.

## 3 DISCUSSION

### 3.A. Advantage of using ontologies and storing data on the World Wide Web

Patients’ data and specifically demographics or clinical details play a crucial role in prediction modeling studies. Transparent and reproducible radiomics research requires availability of data and metadata associated with a particular study. In the case of prediction modeling, these tend to be source images and the clinical outcomes, for example, survival status and survival time interval.

One of the ways to render data FAIR and easily available to be queried remotely over well‐established World Wide Web technology is to archive them as RDF data on a persistent online SPARQL endpoint. This requires existing domain ontologies in order to unambiguously define concepts, and relationships between concepts, by mapping them to standardized terminology. The use of publicly defined ontologies and machine‐readable lexicons overcome the potential barriers of human language understanding and unknown data encodings. The ontologies further apply some level of knowledge representation that follows in the tracks of human logic and inferencing, such that we can use machine‐based queries to discover and process data, without having to first develop extensive knowledge of the relational database structure of the original data. Lastly, we were able to exploit the intrinsically federated pattern matching nature of SPARQL queries to show how to efficiently cross‐reference data from across the clinical, DICOM header, and radiomics domains.

### 3.B. Potential applications

By making this data available on the SPARQL endpoint, we offer a version of the combined DICOM data, clinical information, and radiomics features in a manner that is in closer alignment with FAIR data principles. In this way, we hope to facilitate the investigation of radiomics reproducibility research across different institutions, each of which may speak different human languages, use different imaging protocols, and extract radiomics features in subtly different ways. The queries demonstrated here work in the same way even if these RDF data had been partitioned over multiple databases, irrespective of its geographical location.

As has been shown in other publications, the proposed methodology here can be used prospectively for exchanging radiomics prediction models for training or validation, in accordance with a paradigm known as distributed (or equivalently, federated) machine learning.^41^, ^42^, ^43^

We have provided examples of SPARQL queries, primarily as a form of guidance notes on how to use this data submission. We would encourage the academic community to adjust them according to their own questions and potentially utilize this methodology for multicenter studies. Radiomics researchers that derive immediate benefit from this open resource could be data scientists and medical physicists with some database query experience. Publishing this as a semantic web resource allows real‐time queries and answers about the data. This follows an overall trend toward a growing amount of linked open data with on‐demand access. Online SPARQL tutorials are available.^44^, ^45^, ^46^ We anticipate that the aforementioned audience could build user‐friendly search interfaces on top of this resource, so as to make it more easily used by others with less programming experience.

The reusability of the datasets is strongly supported by the usage of publicly available ontologies, such that the reader is able to look up the ontologies online to search for concepts of interest to them. We have also shared mapping files and RDF conversion scripts on a public code repository, that can also be reused in future.

### 3.C. Limitations of the present submission

One of the major and potentially time‐consuming tasks on the way to publishing the RDF data is the mapping of data fields and data values. We have tried to streamline the process in the current submission by preparing mapping files as templates and, wherever possible, using scripting to control serialization applications such as R2RML. However, it is acknowledged that there is no single universally “correct” mapping to a given target ontology. It is likely that persons working independently could apply the same ontologies but produce quite different (and potentially incompatible) knowledge representations. In the analogy of graphs, there is no single unique graph to represent a given dataset; it is possible to derive many different such graphs that are still logically plausible. In semantic data circles, this is well‐known as the “open‐world” paradigm that is commonly expressed as “anyone can say anything about anything.”

The solution of such a problem is not up to any one piece of investigation nor any one data scientist. As with all conventions and normative standards in healthcare, convergence gradually emerges over time through numerous cycles of usage, refinement, and dissemination. Our methodology and RDF database are therefore not static, so it is intended to be improved and refined together with developing methodology over time.

### 3.D. Possibilities for future development

The question of comparing and then reconciling different data graphs is an ongoing and active line of research in data science. These so‐called shape expressions do not fall within the present scope of submission, but could lead to promising opportunities for improvement. This potentially makes it possible to query data graphs independently of the norms assumed by its publisher.

There is also strong research activity toward stricter standardization of data collection and top‐down imposition of knowledge representation. Unlike the approach used in this work, where we the first had the data and then cast it toward a target ontology, the top‐down approach requires data elements and a data structure to be rigidly defined first of all before the data are collected. This would be very useful for mapping prospective data, but it is less clear how such rigid standards should be applied to legacy data and retrospective studies.

Research is currently in progress toward a modular mapping process, where mappings for generic information that is common for many disease types (e.g., patient demographics) can be rigidly defined and reused often. At the opposite end, highly study‐specific mappings may need to be more dynamic or performed on an ad hoc basis. Modular and piece‐wise reusable mappings for closely related disease types may significantly reduce the overall RDF preparation time, however, at time of writing such a modular process was not yet ready.

## 4 CONCLUSIONS

We have updated and improved four imaging datasets on TCIA. We converted and published clinical data, radiomics features and DICOM headers as online RDF from these four datasets using ontologies and standard web technology. These RDF triples are stored in a public endpoint giving an opportunity to the radiomics community to query these datasets using the SPARQL language. We have demonstrated the realizability of this approach of making the combined data available as FAIR data, in order to incentivize multicenter research into reproducibility of radiomics features across multiple datasets.

Box 1 **Example of a SPARQL query for matching a radiomics feature called “Fmorph.vol” in the IBSI terminology to the overall survival status and survival time of the patients in the LUNG1 collection. Purely for illustrative purposes, we limited the rows of output to 10. The result of the query is shown in Fig. 2.**

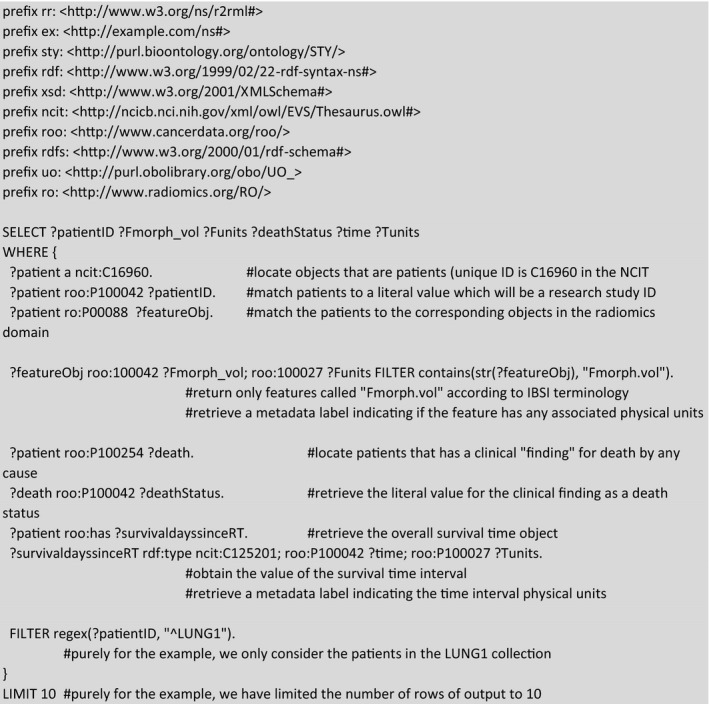

Box 2 **Example of a SPARQL query for examining the different intensity discretization algorithm (i.e., histogram binning) for textural radiomics feature for a single arbitrarily selected subject in the Head–Neck1 collection.**

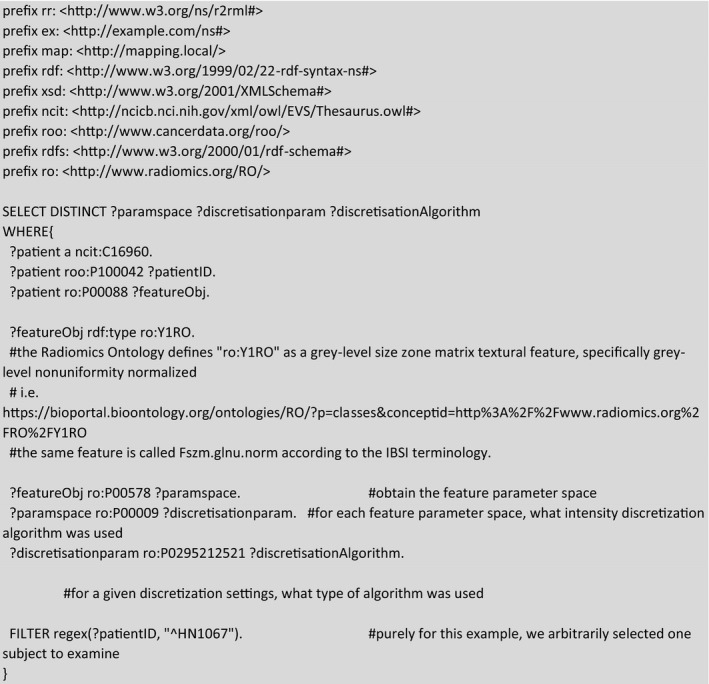

**Fig. 3:**
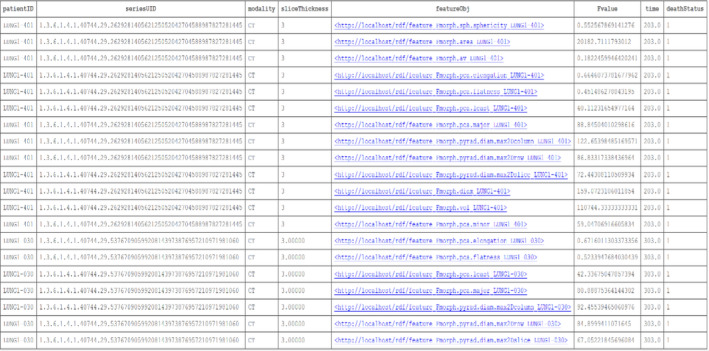
A partial snapshot of the example query given in Box 3. Given as a result of the query are: the subject research ID, the computed tomography series instance unique identifier (UID), the imaging modality and the slice thickness. Each of these are associated with 13 distinct morphological feature concepts (in column featureObj) and the numerical value of each radiomics feature (in column Fvalue). The digital imaging and communications in medicine and radiomics data are cross‐referenced to the vital status and survival time interval as per the example in Box 1. [Color figure can be viewed at wileyonlinelibrary.com]

Box 3 **Example of a SPARQL query for directly cross‐referencing DICOM headers, radiomics features, and survival outcome into a single query. The result of the query is shown in Fig. 3.**

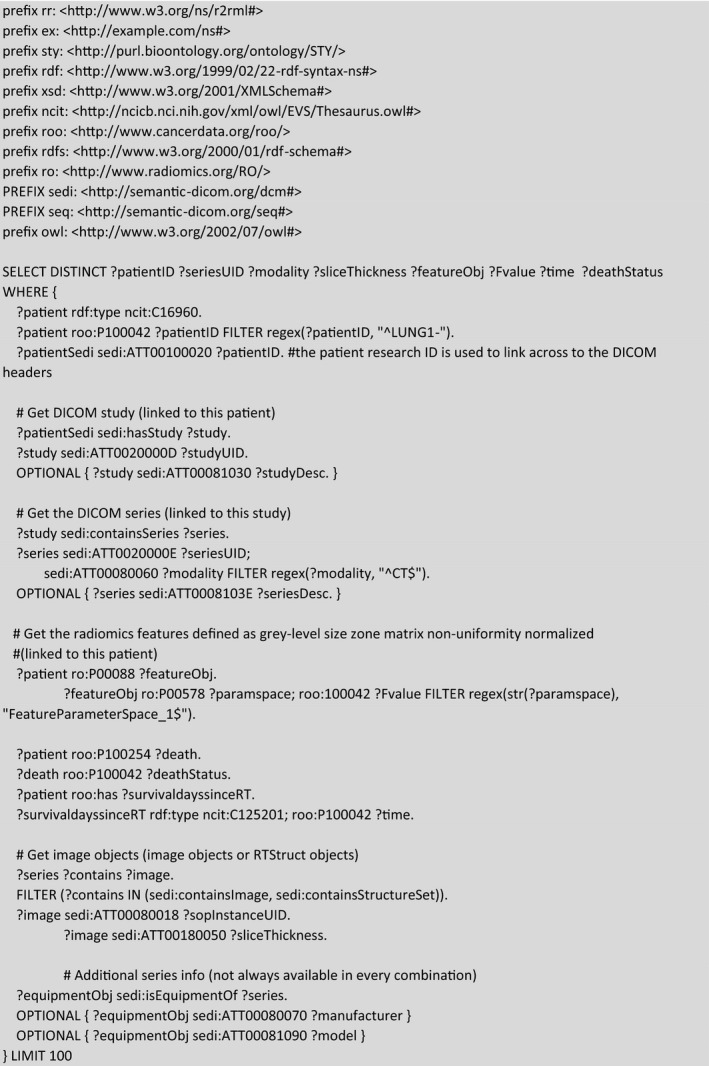

## CONFLICT OF INTEREST

The authors have no conflict of interest to disclose.

5

**Table I:** Overall representation of the datasets previously investigated by Aerts et al^3^. The name of each dataset is accompanied with a URL of The Cancer Imaging Archive collection and a brief summary of the dataset.

Collection	Description
RIDER Lung CT ( link )	This collection was prepared by Zhao et al.^12^ to evaluate the differences of tumor volumetric measurements across “test–retest” CT scans taken at an internal of about 15 min (e.g., a “coffee break”) with the same image acquisition settings. This has been reused for radiomics repeatability and segmentation studies. The associated ROIs denoted *GTVp_test_man* and *GTVp_retest_man* refer to manual delineations in the test and retest series, respectively. The ROIs denoted *GTVp_test_auto* and *GTVp_retest_auto* were initially generated by a semiautomated segmentation algorithm^32^ in the test and retest series, respectively, and manually edited
NSCLC‐Radiomics‐Interobserver1 ( link )	This collection consists of radiotherapy dosimetry planning CT scans of 22 NSCLC subjects treated by conventionally fractionated external beam radiotherapy at a single Dutch center. The ROIs denoted were manually drawn by five experts working independently. The same procedure was repeated after an initial delineation by the above mentioned semiautomatic segmentation algorithm
NSCLC‐Radiomics ( link )	This collection consists of radiotherapy dosimetry planning CT scans of 422 NSCLC subjects treated by conventionally fractionated (chemo)‐radiotherapy at a single Dutch center. The ROI called *GTV‐1* denotes the primary tumor
Head–Neck‐Radiomics‐HN1 ( link )	This collection consists of radiotherapy dosimetry planning CT scans of 137 subjects with either laryngeal or oropharyngeal cancer treated by conventionally fractionated (chemo)‐radiotherapy at a single Dutch center. The ROI called *GTV‐1* denotes the primary tumor
